# Quantifying chlorophylls in melanic lichens: the necessity of separating the absorbance of melanin and chlorophyll

**DOI:** 10.1007/s11120-025-01141-w

**Published:** 2025-02-12

**Authors:** Knut Asbjørn Solhaug, Yngvar Gauslaa

**Affiliations:** Faculty of Environmental Sciences and Natural Resource Management, Norwegian University of Life Sciences, P.O. Box 5003, Ås, NO-1432 Norway

**Keywords:** Absorbance correction, Acetone, *Bryoria*, *Cetraria*, Dimethyl sulfoxide chlorophyll quantification, Extraction methods, Lichens, Melanin

## Abstract

This study investigates extraction and quantification techniques for chlorophylls (Chl) in melanic lichens, with an emphasis on distinguishing between Chl and melanin absorbance during spectrophotometric assessments. We compared various extraction protocols, involving solvents such as dimethyl sulfoxide (DMSO) and acetone, and methods including intact thalli extraction, mortar grinding, and ball mill pulverizing. Three correction methods for melanic absorbance were also compared. Our findings indicated that DMSO was superior for Chl extraction compared to acetone, and differences in efficiency among the DMSO methods were minor. Correction for co-extracted melanin was deemed vital for accurate Chl quantification. The use of a C18 minicolumn was found to be effective for separating Chl and melanic pigments, providing reliable measurements of total Chl and Chl *a/b*-ratios. This method offers a simple cost-effective approach for Chl quantification in extracts containing a mix of Chl and other red light-absorbing pigments.

**Supplementary Information:**

The online version contains supplementary material available at 10.1007/s11120-025-01141-w.

## Introduction

Lichens significantly contribute to biodiversity and play crucial roles in various ecosystems (Ellis 2012; Asplund and Wardle 2017). However, they are increasingly threatened by factors such as forestry practices, global climate change, and chemical pollution including anthropogenic nitrogen deposition (Esseen et al. 2016; Hauck 2011; Ellis 2019; Gauslaa 2024).

Lichens’ ability to convert incoming light into biomass is regulated by the photobiont pool and its photosynthesis. Incoming light is filtered by cortical fungal pigments, such as melanin (Gauslaa and Solhaug 2001), and utilized during moist, active periods (Palmqvist and Sundberg 2000; Dahlman and Palmqvist 2003; Larsson et al. 2014). Chlorophyll (Chl) *a* content serves as a useful indicator of photobiont pools, their photosynthetic capacity, and the growth potential within and across lichen species (Palmqvist and Sundberg 2002; Jansson et al. 2009; Gauslaa et al. 2007). Hence, a reliable determination of Chl is vital for incorporating Chl *a* as a determinant of lichen responses in experimental and environmental monitoring and modelling contexts, and thus for our understanding of lichen performance.

Pigments present in plastids, such as Chl and carotenoids, can be effectively extracted from lichens when wet intact, dry intact, or dry milled. This extraction can utilize a range of solvents including aqueous acetone, ethanol, methanol, dimethyl sulfoxide (DMSO), N,N-dimethylformamide (DMF), and chloroform (Barnes et al. 1992; Arnon 1949). Notably, DMSO and DMF allow extraction without necessitating lichen grinding, while the use of acetone, methanol, ethanol, or chloroform requires grinding in a mortar or milling material (Moran 1982; Hiscox and Israelstam 1979). Following extraction, and if required, centrifugation, the extract’s absorbance is measured at the respective peaks for Chl *a*, Chl *b*, and carotenoids. The concentration of Chl and carotenoids is then calculated using specific formulas based on the solvents used (Arnon 1949; Moran 1982; Porra et al. 1989; Wellburn 1994). It is important to note that these formulas may yield slightly varying results. According to Porra et al. (1989), the formulas proposed by Arnon (1949) may yield highly inaccurate Chl *a/b* ratios and should therefore be avoided.

Despite the effectiveness of DMF in extracting chloroplast pigments without the need for grinding (Moran 1982), its use is generally discouraged due to its high toxicity. DMF is readily absorbed through the skin and can cause liver damage (Kennedy 2012; Li and Zeng 2019). DMSO is preferred because of its lower toxicity and its ability to extract without grinding, much like DMF. It has been regarded superior to acetone for lichens (Barnes et al. 1992; Hiscox and Israelstam 1979). Moreover, a Chl extract in DMSO remains stable for several days (Hiscox and Israelstam 1979; Ronen and Galun 1984; Barnes et al. 1992). The downside to using DMSO lies in its ability to extract other substances that absorb the same red wavelengths as Chl, which can hinder accurate calculation of Chl content in some lichens (Lan et al. 2011). Similarly, when DMSO is used to extract Chl from plants, it sometimes also extracts brown pigments that can distort absorbance measurements, complicating the calculation of Chl (Minocha et al. 2009). For lichens, the presence of melanin becomes an issue due to its effective extraction by DMSO (Meeßen et al. 2013). However, existing protocols for measuring Chl in lichens do not account for potential interference from melanin (Barnes et al. 1992; Palmqvist and Sundberg 2002). This is noteworthy given that a significant number of lichens are melanic (Brodo and Hawksworth 1977; Esslinger 1977; Rikkinen 1995). The issue of melanin interference also extends to the use of DMF for Chl extraction, because melanins are soluble in DMF (Deepthi et al. 2021).

Beyond the potential interference with Chl quantification, the exploration and development of methods for isolating and quantifying melanin presents intriguing ecological and physiological prospects. Melanic pigments are particularly prevalent in lichens that inhabit environments with high abiotic stress (Gostinčar et al. 2012), with their synthesis often triggered by UV, specifically UV-B (Gauslaa and Solhaug 2001; Solhaug et al. 2003).

The chemical composition and biosynthetic pathways of lichen melanins, however, remain largely unknown (Mafole et al. 2019). It has been observed that melanic pigments from lichens in the Peltigerales order, such as *Lobaria pulmonaria*, belong to the DOPA melanins (Matee et al. 2016). Conversely, the dark pigments from lichens in the Lecanorales, including *Bryoria* and *Cetraria*, display characteristics typical of allomelanins (Mafole et al. 2019). Interestingly, DOPA melanins contain significantly more N than allomelanins, probably because members of the Peltigerales, unlike those in the Lecanorales, readily access N through their symbiotic N_2_-fixing cyanobacteria. For a more comprehensive discussion on melanic pigments in lichens, refer to Mafole et al. (2019).

Our first objective is to explore methods for Chl extraction and identify effective and time-saving extraction protocols by comparing extractions of (1) intact thalli, after (2) grinding in a mortar or (3) ball mill pulverizing for both dry and moist lichens. Additionally, we will examine the effect of ultrasonic treatment. Lastly, we will compare extractions using DMSO and acetone.

The second objective is to discern techniques effective in adjusting for the absorbance of melanic pigments extracted with DMSO. Our approach involves comparing three correction methods: Firstly, we will separate melanins and Chl using a C18 minicolumn (Chowdhury et al. 2017). Secondly, we will employ the techniques of subtracting the absorbance of a commercial melanin spectrum. Lastly, we will estimate melanin absorbance in the Chl absorbance region by extrapolating the linear regression of the sample’s own absorbance between 710 and 780 nm to the relevant lower wavelengths. Upon completion of these steps, our last task will be to authenticate the spectroscopically determined Chl values by comparing them with values obtained from HPLC analyses.

## Materials and methods

### Lichen material

We collected *Cetraria islandica* Ach., 1100 m a.s.l. (61.36 N, 10.12 E) and *Bryoria fuscescens* 600 m a.s.l. (61.44 N, 10.41 E) from Ringebufjellet, eastern Norway in late summer (August 21st 2022), henceforth referred to by genus names only. New specimens for HPLC validation experiments were collected at the same site August 12th 2024. The lichens were stored in a refrigerator until the time of analysis.

### Extraction methods

The two species, *Cetraria* and *Bryoria*, significantly differed in their Chl concentration. Therefore, more material was needed for Chl extraction from *Cetraria*. The detailed extraction methods are elaborated below and summarized in Table 1. In all extraction methods, MgCO_3_ was added to DMSO and acetone to prevent possible degradation of Chl into pheophytin.

**Table 1:** Extraction protocols of lichen material

Solvent	Lichen treatment	Extraction time	Temperature	Ultrasonic bath
DMSO	Milled, dry	30 min	60 °C	No
DMSO	Ground in mortar	15 min	20 °C	No
DMSO	Milled, dry	24 h	20 °C	No
DMSO	Intact, wet	24 h	20 °C	No
DMSO	Intact dry	24 h	20 °C	No
DMSO	Milled, dry	30 min	60 °C	Yes
Acetone	Ground in mortar	15 min	20 °C	No
Acetone	Milled, dry	24 h	20 °C	No

DMSO and acetone were MgCO_3_−saturated to avoid possible Chl degradation

1. The thalli were finely ground using a ball mill. Pigments were extracted from approximately 30 mg of dry, pulverized *Cetraria* thalli and 15 mg *Bryoria* thalli with 1.5 mL DMSO at 60 °C for 30 min in a 2 mL Eppendorf tube.
2. Pigments were extracted from roughly 100 mg of dry thalli by thoroughly grinding in a mortar at 20 °C with a small quantity of DMSO. The mortar was rinsed twice with DMSO, and the combined sample volume was adjusted to 10 mL for *Cetraria* and 20 mL for *Bryoria*.
3. The thalli were finely ground using a ball mill. Pigments were extracted from approximately 30 mg of dry, pulverized thalli from *Cetraria* and 15 mg from *Bryoria* with 1.5 mL DMSO at 20 °C for 24 h in a 2 mL Eppendorf tube.
4. Approximately 30 mg of dry, intact thalli from *Cetraria* and 15 mg from *Bryoria* were moistened with de-ionized water. Pigments were extracted with 1.5 mL DMSO in a 2 mL Eppendorf tube for 24 h at 20 °C.
5. Pigments were extracted from approximately 30 mg of dry, intact thalli from *Cetraria* and 15 mg from *Bryoria* with 1.5 ml DMSO in a 2 mL Eppendorf tube for 24 h at 20 °C.
6. Thalli were finely ground using a ball mill. Pigments were extracted from approximately 30 mg of dry, pulverized thalli from *Cetraria* and 15 mg from *Bryoria* with 1.5 mL DMSO in a 2 mL Eppendorf tube for 30 min at 60 °C in an ultrasonic bath.
7. Pigments were extracted from approximately 100 mg of dry thalli by thoroughly grinding in a mortar at 20 °C using a small volume of 80% acetone. The mortar was rinsed twice with acetone, and the combined sample volume was adjusted to 10 mL for *Cetraria* and 20 mL for *Bryoria*.
8. Thalli were finely ground using a ball mill. Pigments were extracted from approximately 30 mg of dry pulverized thalli from *Cetraria* and 15 mg from *Bryoria* with 1.5 mL acetone in a 2 mL Eppendorf tube for 24 h at 20 °C.

Extracts derived from intact thalli were transferred into Eppendorf tubes and centrifuged. Extracts of milled samples were centrifuged. The absorbance of all extracts was measured from 350 to 800 nm using a Shimadzu UV-2101 spectrophotometer (Shimadzu, Kyoto, Japan) with a slit width of 1 nm. The spectra were normalized to represent 10 mg dry matter (DM) in 1 mL solvent.

All DMSO extracts contained brown melanic pigments. We used a silica Agilent Bond Elut C18 column (Agilent Technologies, Inc, USA) to separate melanin and Chl. Initially, we conditioned the column by forcing 0.5 mL of DMSO through it. Afterwards, 1 mL of the previously obtained lichen extract was passed through the column. To ensure the complete elution of melanic pigments, an additional 0.5 mL of pure DMSO was added to the column. The two DMSO fractions were then combined. The absorbance of this melanic fraction was measured from 350 to 800 nm and normalized to represent 10 mg DM in 1 mL solvent. Before reusing the column, the silica-bound Chl was removed using 0.5 mL of 100% acetone. This acetone fraction was not employed for chlorophyll estimation due to its composition being a mixture of DMSO and acetone, for which a suitable formula is unavailable. However, as a test, the acetone fraction was measured for 5 thalli of *Bryoria* using formulas for 100% acetone (Wellburn 1994).

### Correction methods

**Fig. 1:**
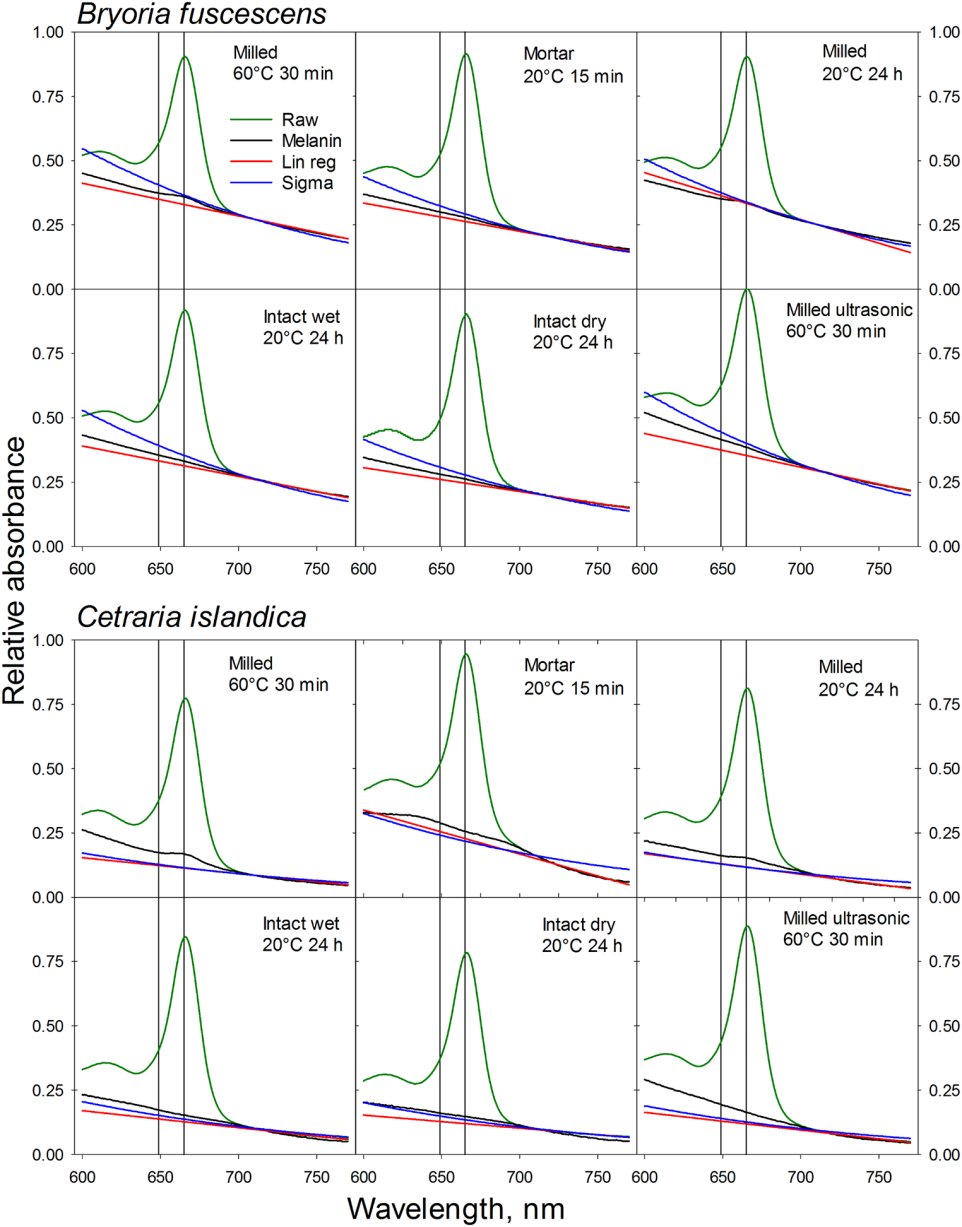
Spectra of raw DMSO extracts of chlorophyll from *Bryoria fuscescens* and *Cetraria islandica* (green lines) with different extraction methods. Correction spectra with lichen melanin extract (black lines), Sigma melanin (blue lines) and with linear regression correction from the raw extract extrapolated from 770 to 710 nm (red lines) are shown. The correction spectra for lichen melanin and Sigma melanin are normalized to 710 nm absorbance of the raw extract

The following methods were implemented to correct for the presence of melanin in the raw Chl extracts:

1. The melanin spectrum was normalized to the same absorbance at 710 nm as the raw Chl spectrum before it was subtracted from the raw Chl spectrum (see Fig. 1). Subtraction of the melanin spectrum from the raw spectrum without normalization might introduce errors due to minor inaccuracies in volumes.
2. A linear regression line, extrapolated from the chlorophyll extracts’ spectrum between 770 and 710 nm, was used to determine correction factors needed for the values at 665 and 649 nm in the original Chl spectrum (see Fig. 1).
3. A synthetic Sigma melanin (M8631, Lot #BCBV7256 Pcode102078508; SIGMA-Aldrich CAS-number 8049-97-6) spectrum was adjusted to match the DMSO Chl spectrum’s absorbance at 710 nm. Thereafter, the respective absorbances at 649 and 665 nm of the melanin spectrum were subtracted from the DMSO Chl spectrum (see Fig. 1).

After applying these different correction protocols, the concentration (in µg mL^− 1^) of Chl *a* and *b* was calculated using Wellburn’s (1994) formulas (Table 2).

**Table 2:** Equations used to calculate Chl *a* and *b* in DMSO and acetone

DMSO extracts	Acetone extracts
Chl *a* = 12.19A_665_ – 3.45A_649_	Chl *a* = 12.21A_663_ – 2.81 A_646_
Chl *b* = 21.99A_649_ – 5.32A_665_	Chl *b* = 20.13A_646_ – 5.03A_663_

### Validation of extraction efficiency

**Fig. 2:**
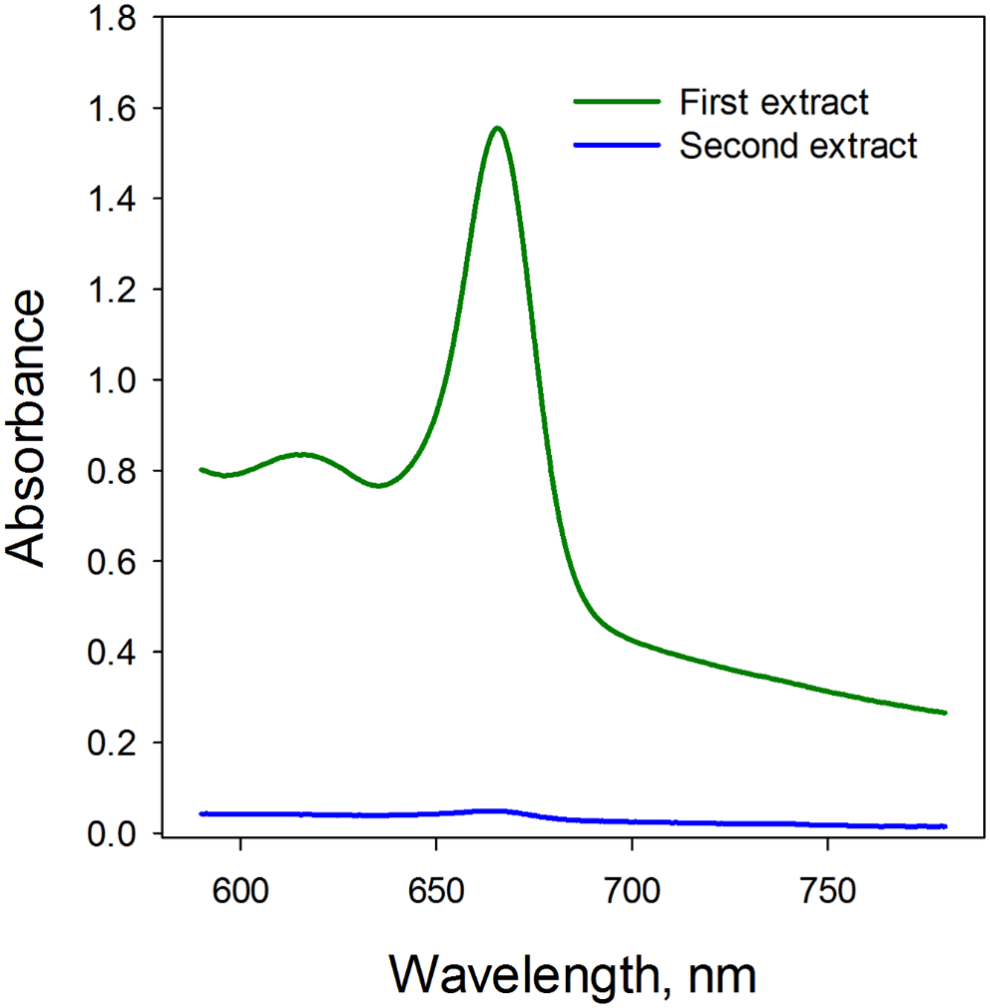
The absorbance spectrum for the DMSO extract of *Bryoria fuscescens* extracted for 24 h in darkness at room temperature (green line) and of the same thalli reextracted for additional 24 h (blue line). Each line represents the average value of five thalli

Pigments were initially extracted from 10 to 15 mg dry, intact *Bryoria* thalli over a period of 24 h using 1.5 mL DMSO in Eppendorf tubes. This was followed by a re-extraction process for another 24 h. As indicated by the absorbance spectra for both extracts, shown in Fig. 2, nearly all Chl and the extractable portion of melanin were successfully extracted during the initial 24 h period.

### Efficiency of melanin extraction with DMSO compared with NaOH extraction

Pigments were extracted from intact *Bryoria* thalli in 1.0 M NaOH in Eppendorf tubes for 24 h at room temperature. For comparison, pigments were extracted also from similar thalli with DMSO under identical conditions. The melanins were then separated from the DMSO extract using a C18 minicolumn. Absorbance spectra from 400 to 800 nm were measured.

### Validation of chlorophyll correction with HPLC

The analysis of chlorophylls was carried out using an Agilent Technologies 1200 Series HPLC system (Waldbronn, Germany). The system was furnished with a binary pump (G1312A), an autosampler (G1329A), a column oven (G1316A) and a diode array detector (DAD) (G1315D). For the separation of chlorophylls, a Thermo Scientific (ODS Hypersil 250 × 4.6 mm) column was employed. The injection volume was set at 20 µL with a solvent flow rate of 1.2 mL min-^1^. The analysis was initiated with a linear gradient of 100% A (acetonitrile: methanol: water (84:9:7) v/v/v and TRIS-HCl buffer 10 mM pH 8) to 100% B (methanol: ethyl acetate (68:32) v/v) for 12 min. This was succeeded by isocratic elution with 100% B for the next 6 min. Afterwards, a linear gradient was applied, shifting from 100% B to 100% A within 1 min before the column was equilibrated with 100% A for 6 min in preparation for the next injection (Fernández-Marín et al. 2018). Chlorophyll calibration was conducted by extracting five spinach leaf disks using DMSO. The concentrations of chlorophyll *a* and *b* in these extracts were calculated according to Wellburn (1994). These extracts were then injected into the HPLC and response factors for the peak areas of chl *a* and *b* were determined. Finally, DMSO extracts from intact dry *Cetraria* and *Bryoria* were injected into the HPLC, and chlorophylls were estimated using the spinach response factors.

### Statistical methods

We employed General Linear Models (GLM) to analyze (1) the total Chl concentration and (2) the Chl *a/b* ratio. The fixed factors were the lichen species, extraction method, and correction method. For comparison of different correction methods with HPLC analysis and acetone fraction we used one-way ANOVA followed by Tukey multiple comparison test. We conducted these analyses in Minitab^®^ version 22.1 (Minitab Inc., State College, PA, USA) software. To meet the requirements of the models, we applied a Box-Cox-transformation to the data.

## Results

### Validation of chlorophyll correction using HPLC

A comparison of Chl concentrations and *a/b* ratios in DMSO extracts analyzed with HPLC, along with estimates from various correction methods, is presented in Table 3. The HPLC analysis confirmed that the correction methods were generally effective for total Chl quantification. For Chl *a/b* ratios, the correction methods performed well for *Cetraria*, which is characterized by moderate melanin content. However, the correction method using normalized melanin yielded the best results for the highly melanic *Bryoria* (Table 3). Furthermore, the presence of pure, single peaks for Chl *a* and *b* in the HPLC chromatograms indicates low or no degradation of chlorophyll during 24 h DMSO extraction (Supplementary Material, Fig. S1).

**Table 3:** Comparison of different correction methods with HPLC analysis of chlorophyll extracted with DMSO from dry, intact lichens

Method		Raw extract	HPLC	Corrected linear regression	Corrected Sigma melanin	Corrected normalized melanin
*Bryoria* *fuscescens*	Chl mg g-1	3.46 ± 0.24^a^	1.53 ± 0.14^b^	1.73 ± 0.12^b^	1.50 ± 0.13^b^	1.56 ± 0.14^b^
	Chl *a/b*	1.25 ± 0.09^d^	5.19 ± 0.18^b^	3.72 ± 0.22^c^	7.38 ± 0.31^a^	5.75 ± 0.33^b^
*Cetraria* *islandica*	Chl mg g-1	0.70 ± 0.06^a^	0.53 ± 0.08^a^	0.63 ± 0.07^a^	0.63 ± 0.07^a^	0.63 ± 0.07^a^
	Chl *a/b*	2.56 ± 0.17^a^	3.64 ± 0.27^b^	3.74 ± 0.16^b^	3.74 ± 0.16^b^	3.86 ± 0.17^b^

The values represent the average of five replications. Different letters indicate significant difference at the 5% level, as determined by one-way ANOVA and Tukey’s *post hoc* test

### Extraction methods

**Fig. 3:**
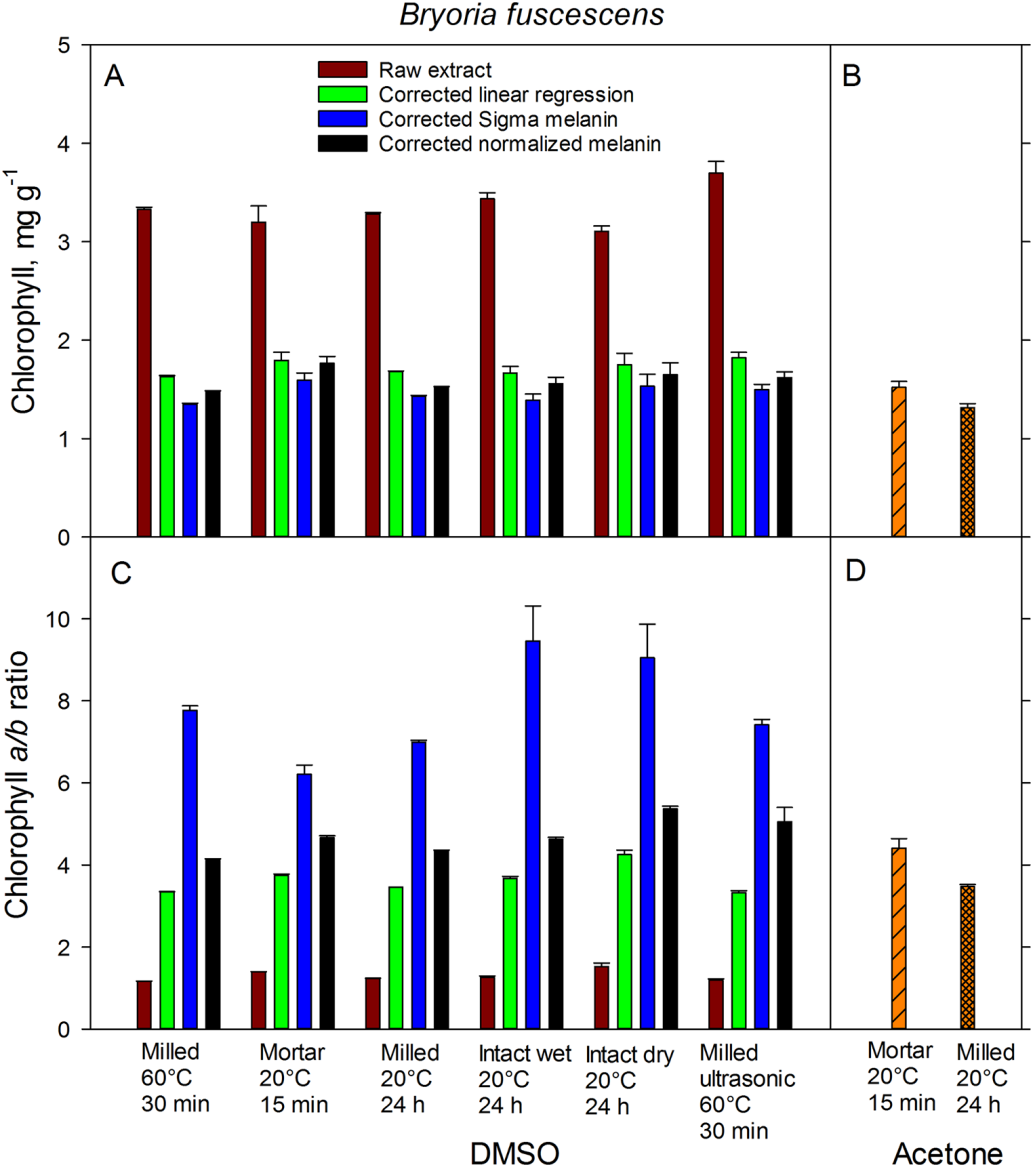
Total chlorophyll concentration (**A**, **B**) and chlorophyll *a/b* ratio (**C**, **D**) in the hair lichen *Bryoria fuscescens* extracted in DMSO (**A**, **C**) and acetone (**B**, **D**) using various grinding techniques, extractions methods, and extraction times. Brown bars show values not corrected for melanin; green, blue, and black bars show values corrected for melanin absorbance with linear regression, Sigma melanin, and with normalization, respectively. Error bars show ± 1 SE (*n* = 5)

**Fig. 4:**
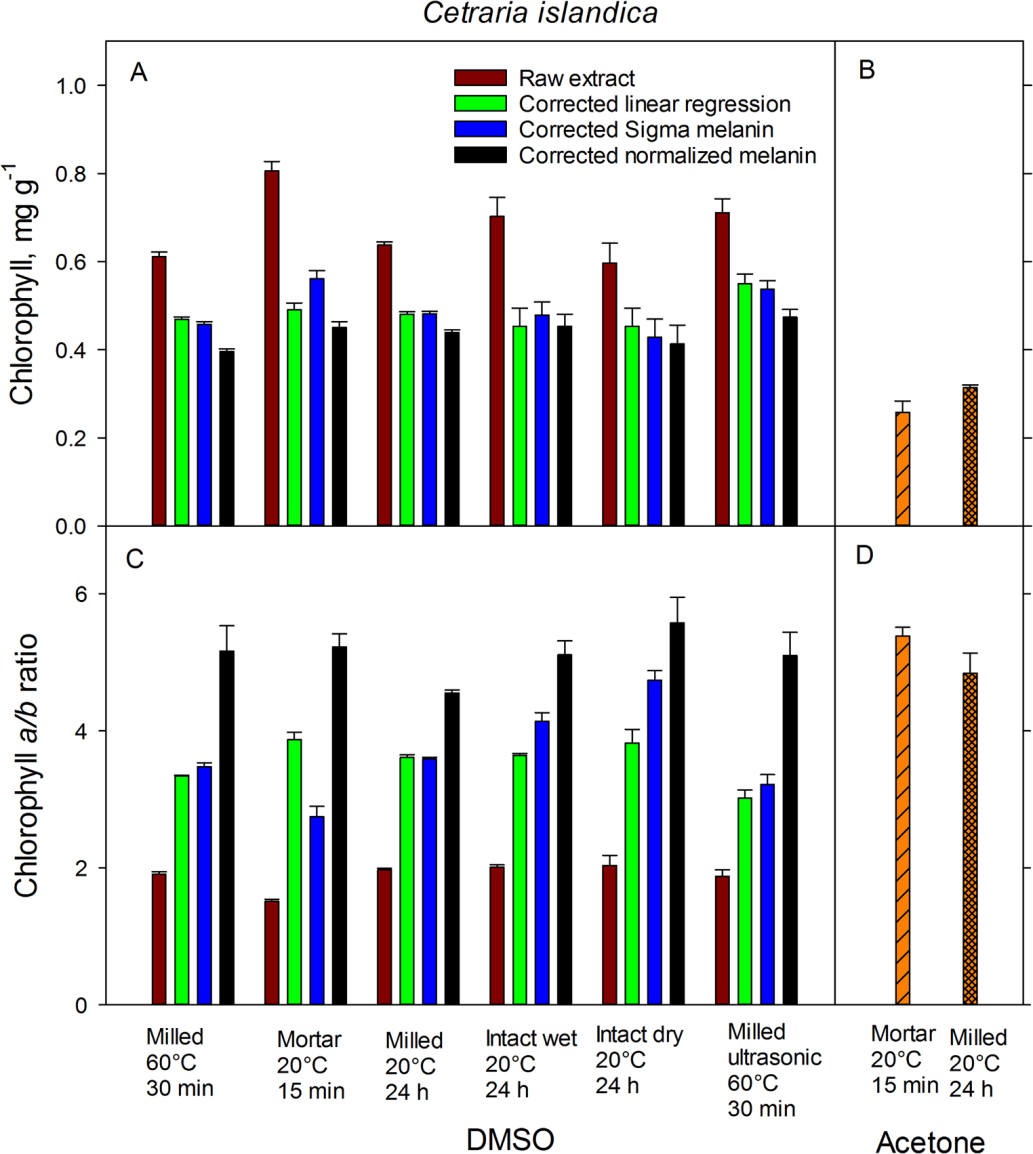
Total chlorophyll concentration (**A**, **B**) and chlorophyll *a/b* ratio (**C**, **D**) in the mat-forming lichen *Cetraria islandica* extracted with DMSO (**A**, **C**) and acetone (**B**, **D**) using various grinding techniques, extractions methods, and extraction times with. Brown bars show values not corrected for melanin; green, blue, and black bars show values corrected for melanin absorbance with linear regression, Sigma melanin and with normalization respectively. Error bars show ± 1 SE (*n* = 5)

The variation in the total Chl concentrations and Chl *a/b* ratios was largely attributed to lichen species (*P* < 0.001), although the extraction method remained a significant (*P* < 0.001) factor in a three-way GLM (Table 4). *Bryoria* showed a Chl concentration more than three times as high as that of *Cetraria* (Figs. 3 and 4). The interaction between factors of species and extraction method was weakly significant (*P* = 0.013) for the Chl concentration model, and not significant for the Chl *a/b* ratio model (*P* = 0.068).

**Table 4:** General linear models for total Chl and the Chl *a/b* ratio in two melanic lichens (*Bryoria fuscescens* and *Cetraria islandica*) using six extraction methods with DMSO and three correction methods

Parameter	Total chlorophylls; *R*^2^_adj_ = 97.36			Chl a/b-ratio; *R*^2^_adj_ = 91.42	
	df	F	*P*	F	*P*
Species (S)	1	6488.49	< 0.001	295.45	< 0.001
Extraction (E)	5	8.56	< 0.001	28.89	< 0.001
Correction (C)	2	16.79	< 0.001	312.96	< 0.001
S x E	5	3.01	0.013	2.10	0.068
S x C	2	17.83	< 0.001	364.94	< 0.001
E x C	10	0.63	0.068	12.98	< 0.001
Error	154				
Total	179				

Mean values for the various treatments analyzed by the GLM are given in Figs. 3 and 4. The S x E x C interaction was not significant and therefore excluded in the final model. Data from the raw extract and from acetone extraction were not included in the GLM because of unreliable results and incomplete extraction, respectively

For the species *Bryoria*, the highest total Chl concentrations were obtained by an ultrasonic treatment for 30 min at 60 °C or by grinding intact dry samples in a mortar. In contrast, the lowest values were found in samples that were milled (Fig. 3). Similarly, in the case of the species *Cetraria*, the highest Chl concentrations were found in samples extracted with ultrasonic treatment and mortar grinding (Fig. 4). Conversely, the lowest Chl concentrations were found in intact dry samples and those subjected to a 30-min extraction at 60°. Despite these differences, the variation between methods was low. However, a significant variation was noticed in the Chl *a/b* ratios across the different extraction methods for both species, particularly for *Bryoria* (Fig. 3). This variation was amplified in samples corrected with Sigma melanin (Fig. 1), due to its high absorbance at 665 and 649 nm. This high absorbance can be attributed to the fact that the absorbance of Sigma melanin increases more with decreasing wavelength than that of melanin extracted from lichens (Fig. 1). The standard error for both Chl concentration and Chl *a/b*-ratio was significantly reduced for the milled samples of both species. As replicates for milled samples were taken from the same sample, there was no individual difference. However, for intact lichens, separate thalli were used as replicates. This implies that the variation observed in extraction methods with intact thalli could be attributed to an individual difference between the specimens.

### Correction methods

We observed significant variation in the Chl *a/b* ratios across the different correction methods. In the case of *Bryoria*, the Sigma melanin demonstrated a significantly greater absorbance correction compared to the correction done with melanic pigments from lichens and linear regression correction. This effect was more pronounced at shorter wavelength and could significantly impact the estimation of Chl *a/b* ratios (Fig. 1). Consequently, the Sigma correction led to a slightly lower total Chl concentration and exceptionally high Chl *a/b* ratios. Conversely, for *Cetraria*, the Sigma melanin correction was lower than correction with melanic pigments from the lichen. This resulted in marginally higher Chl concentration and lower Chl *a/b* ratios. The contrasting effect of the Sigma melanin correction led to a highly significant interaction between the factors of species and correction method for both Chl concentration and Chl *a/b* ratio (Table 5).

**Table 5:** Comparison of various correction methods for chlorophyll extracted using DMSO from dry, intact *Bryoria fuscescens* with estimation of chlorophyll eluted with acetone from the C18 mini column

Method		Raw extract	Corrected linear regression	Corrected Sigma melanin	Corrected normalized melanin	Acetone fraction
Chl mg g^− 1^		3.47 ± 0.42^a^	1.95 ± 0.18^b^	1.71 ± 0.15^b^	1.86 ± 0.20^b^	2.10 ± 0.11^b^
Chl a/b		1.54 ± 0.13^c^	4.02 ± 0.21^b^	7.98 ± 0.98^a^	4.80 ± 0.08^b^	3.50 ± 0.29^bc^

The data presented are an average of 5 replicated trials, The presence of different letter signifies a significant difference at 5% level analyzed with one-way ANOVA and Tukey *post hoc* test

### Extraction solvent

The extraction of Chl with milled samples was more efficient when using DMSO as compared to acetone for both species under study. However, in the case of *Bryoria*, the efficiency was equal when using either acetone or DMSO, as long as the samples were ground in a mortar (Figs. 3 and 4). The best correction for Chl estimation using DMSO as a solvent can be achieved by deducting the lichen melanin absorbance. When compared to DMSO, acetone extracted slightly less Chl from *Bryoria* and significantly lower extraction from *Cetraria*, regardless of whether the samples were milled or ground with a mortar (Figs. 3 and 4). Notably, no Chl was extracted from intact thalli with acetone (data not shown). The lower Chl extraction efficiency from *Cetraria* compared to *Bryoria* when using acetone could be attributed to the harder and more difficult-to-grind nature of *Cetraria* thalli. Importantly, acetone did not extract any brown melanic pigments.

One might consider deducting the baseline absorbance at 750 nm, assuming equal non-Chl absorbances at 649 and 665 nm. However, note that the absorbance of the melanic pigments, which pass through the C18 column, tends to increase as the wavelength decreases (refer to Fig. 1). The absorbance of pure Sigma melanin also increases with decreasing wavelength (Fig. 1). Therefore, deducting the 750 nm baseline absorbance, a procedure sometimes used for slightly cloudy extracts, may lead to significant inaccuracies.

### Estimation of chlorophyll in the acetone extract from the C18 minicolumn

The total amount of chlorophyll was not significantly different in the acetone fraction from the minicolumn (after separation of melanin) and after applying the three other correction methods. The chl *a/b* ratio was much higher when corrected with Sigma melanin, whereas the values were not significantly different between acetone fraction and the two other methods (Table 5).

### Comparison of melanin extraction with DMSO and 1.0 M NaOH

**Fig. 5:**
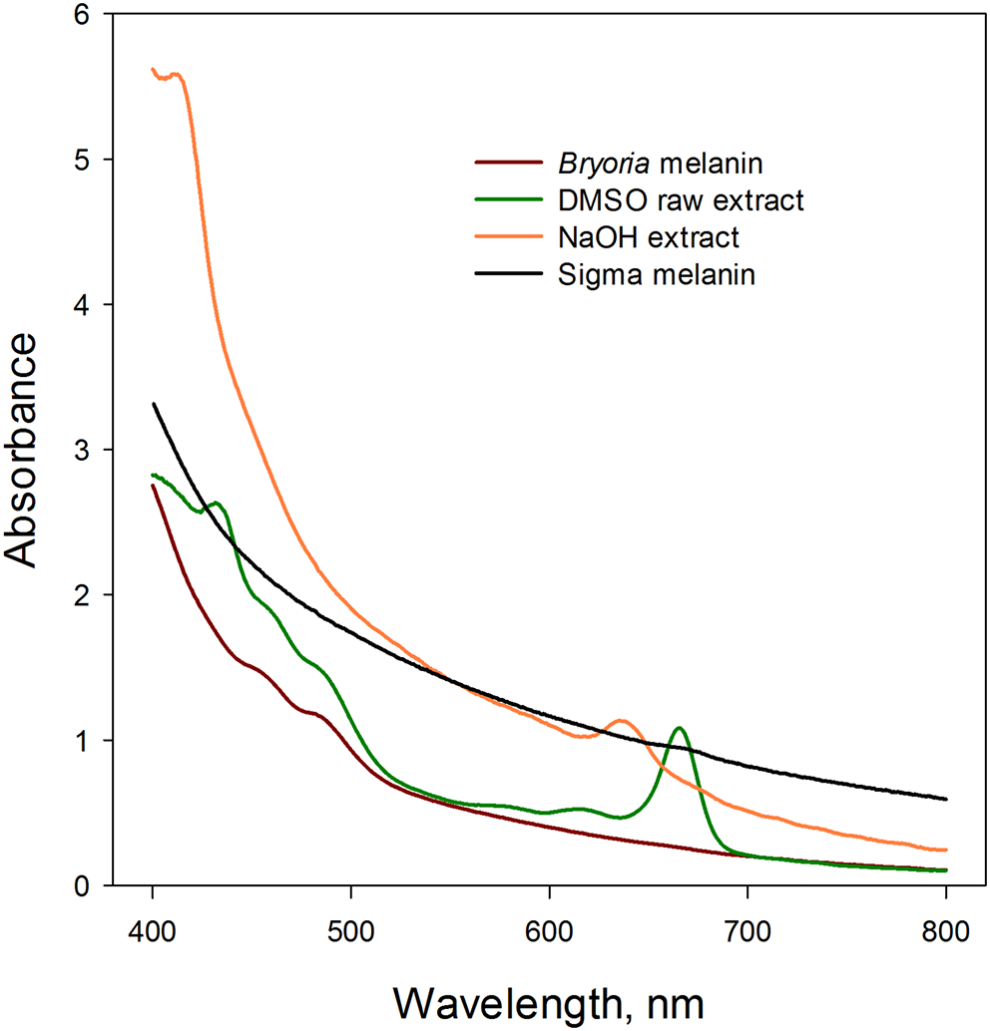
Absorbance spectra of *Bryoria* DMSO raw extract, *Bryoria* melanins separated with the C18 column, *Bryoria* melanin extracted with 1.0 M NaOH and Sigma melanin. *Bryoria* melanin and the DMSO raw extract spectra are normalized to equal values at 710 nm, and the NaOH extract spectrum represent the absorbance of the same dry mass as used for DMSO. Each spectrum is the average of three replicates

The extraction of melanins from *Bryoria* thalli is significantly more effective with NaOH compared to DMSO (Fig. 5). Absorbance at 710 nm of the NaOH extract was 150% higher than that of the DMSO extract. In addition, the thalli were partly broken down by the NaOH treatment.

## Discussion

### Estimation of Chl without melanin correction

Comparing Chl concentrations and Chl *a/b* ratios in DMSO extracts from melanic lichens analyzed using HPLC with values calculated directly from absorbance of the raw extracts reveals significant discrepancies. Specifically, equations without correction yield excessively high concentrations and unusually low a/b ratios (Table 3). This discrepancy is attributed to the extraction of melanic pigments from both *Bryoria* and *Cetraria* with DMSO, leading to an absorbance spectrum that includes Chl, melanin, and other pigments (Fig. 1). The Chl concentration is calculated by equations (e.g., Wellburn 1994), which use the absorbances in the red region of the spectrum where other chloroplast pigments like carotenoids do not absorb light. However, melanic pigments also strongly absorb red light. To account for this, a baseline absorbance above 710 nm – a range where Chl does not absorb light – needs to be subtracted. This is due to the escalating absorbance of both synthetic melanin and melanic pigments separated with the C18 column as the wavelength decreases (Fig. 1). Failing to adjust for melanin absorbance can therefore lead to a significant overestimation of Chl concentration. Simultaneously, the estimated Chl *a/b* ratio can be considerably underestimated due to a larger relative increase at the 649 nm Chl *b* absorbance peak compared to the maximum Chl *a* absorbance at 665 nm. Despite DMSO’s superior extraction efficiency (Lan et al. 2011), it is not the ideal solvent for extracting Chl from lichens with melanic compounds due to this overlap in absorbance. The use of a C18 column mitigates this problem by effectively separating melanin from Chl, thereby facilitating accurate estimates of Chl and Chl *a/b*-ratio.

### Extraction methods

The efficiency of total Chl extraction showed minor but significant variations between the different methods used for *Bryoria* and *Cetraria*. Extraction from intact lichens required relatively longer extraction times for complete Chl extraction. However, the constant total Chl content and Chl *a/b* ratios in DMSO over time indicate minimal Chl degradation. In addition, the presence of clear, single peaks for Chl *a* and *b* (Fig. S1) indicates low degradation of chlorophyll after extraction for 24 h at room temperature.

If fast extraction is needed, the sample can be heated in DMSO for 30 min at 60 °C. This method showed no signs of chlorophyll degradation when compared to fast extraction at room temperature using a mortar. According to Ronen and Galun’s study (1984), heating at 65 °C for 40 min accelerates the extraction process. Post this, the DMSO Chl extract can be stored at 0–4 °C for several days without any degradation (Barnes et al. 1992; Hiscox and Israelstam 1979).

The extraction of intact dry lichen thalli is less labour-intensive. Total Chl content and Chl *a/b* ratios were found to be similar after extraction from dry intact thalli, milled thalli, or thalli ground in a mortar.

### Correction methods

In principle, separating melanin from Chl in extracts should yield the most accurate results for both total Chl and Chl *a/b* ratio. Our use of different correction methods resulted in larger variations in Chl *a/b* ratios than in total Chl contents. This is because the error in the absorbance correction is more pronounced at the Chl *b* absorbance peak (649 nm) compared to the Chl *a* peak (665 nm). The estimation of total chlorophyll content primarily relies on the Chl *a* peak at 665 nm (Table 2), whereas the Chl *a/b* ratio is more dependent on Chl *b* content and thus the 649 nm absorbance (Table 2).

Alhough acetone does not completely extract Chl, we can assume that the Chl *a/b* ratios are accurate because acetone does not extract melanic pigments that absorb light at 649 and 665 nm. The fact that we found similar Chl *a/b* ratios in DMSO extracts corrected for extracted melanin, as in acetone extracts, indicates that the correction for melanin separated with the C18 column provides accurate Chl *a/b* ratios.

Melanins represent a diverse chemical group, with different lichen species containing specific types of melanins such as N-rich eumelanins and N-poor allomelanins (Mafole et al. 2019). The varied absorbance spectra of these melanic compounds may account for the discrepancy in correction when using synthetic Sigma melanin for *Bryoria* and *Cetraria*, and could explain why synthetic melanin may yield inaccurate results. The extraction efficiency of melanin using DMSO is significantly lower compared to an alkaline solution like NaOH, which is the recommended extraction method for melanins (Pralea et al. 2019). This is evident in Fig. 5, indicating that melanin extraction with DMSO is incomplete. Furthermore, the negligible melanin content in the additional DMSO extraction (Fig. 2) suggests that most freely soluble melanin is extracted during the first extraction with DMSO. However, melanins may be tightly bound to proteins (see e.g. Pralea et al. 2019). For instance, in the lichen *Lobaria pulmonaria*, melanins could be firmly bound to hyphal cell wall components (Daminova et al. 2023). This suggests that while a fraction of the melanins can be easily extracted with DMSO, the extraction of more tightly bound melanins requires a strong alkaline solution.

Additionally, the C18 minicolumn can be highly beneficial for measuring Chl in various types of photosynthetic materials, particularly when other pigments with overlapping absorbances spectra are also extracted.

## Conclusion

Our study revealed minor differences in extraction efficiency across various methods utilizing DMSO. The physical state of the thalli (intact, milled, or ground), and whether it was in a wet or dry state, did not significantly impact the results. However, it was clear that acetone was less efficient than DMSO, aligning with earlier observation (Hiscox and Israelstam 1979; Barnes et al. 1992).

While DMSO is an efficient solvent for extracting Chl, it is essential to correct for co-extracted compounds when dealing with melanic lichens. Although High-Performance Liquid Chromatography (HPLC) is likely the most precise method for the separation and analysis of Chl, the use of a cost-effective C18 minicolumn also yielded reliable results for total Chl, Chl *a/b* ratios. Corrections using linear regression from 780 to 710 nm or synthetic commercial melanin provided reasonably accurate total Chl concentrations, though the Chl *a/b* ratios were not accurate. Therefore, we recommend extracting Chl using DMSO and separate the brown pigments and Chl using a C18 column for Chl determination in melanic lichens. This method is particularly beneficial when dealing with a large sample size, as HPLC could be too time-consuming and expensive.

## Electronic supplementary material

Below is the link to the electronic supplementary material.

Supplementary Material 1

## Data Availability

No datasets were generated or analysed during the current study.
