# Supplementary material for: Quantifying chlorophylls in melanic lichens: the necessity of separating the absorbance of melanin and chlorophyll
Source: Photosynth Res. 2025 Feb 12;163(1):17. doi: 10.1007/s11120-025-01141-w (PMC11821740; doi:10.1007/s11120-025-01141-w)
Supplement: Supplementary file 1 — Supplementary Material 1 [file 11120_2025_1141_MOESM1_ESM.pdf]

Cetraria islandica

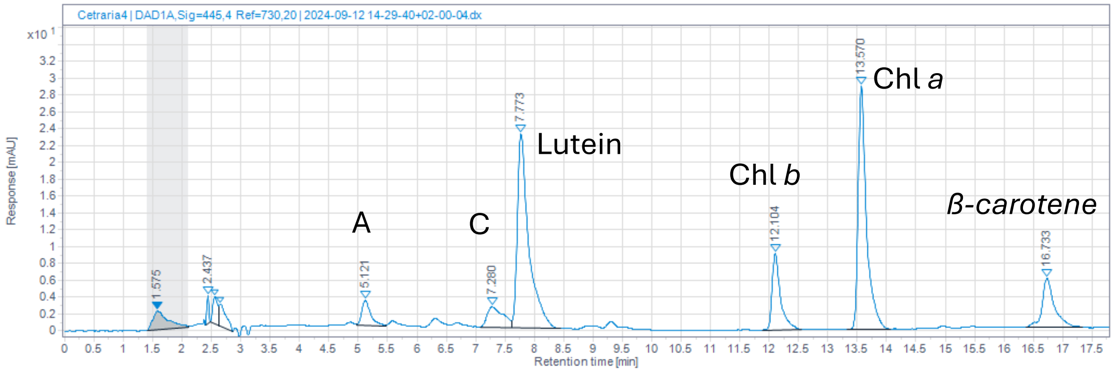

Bryoria fuscescens

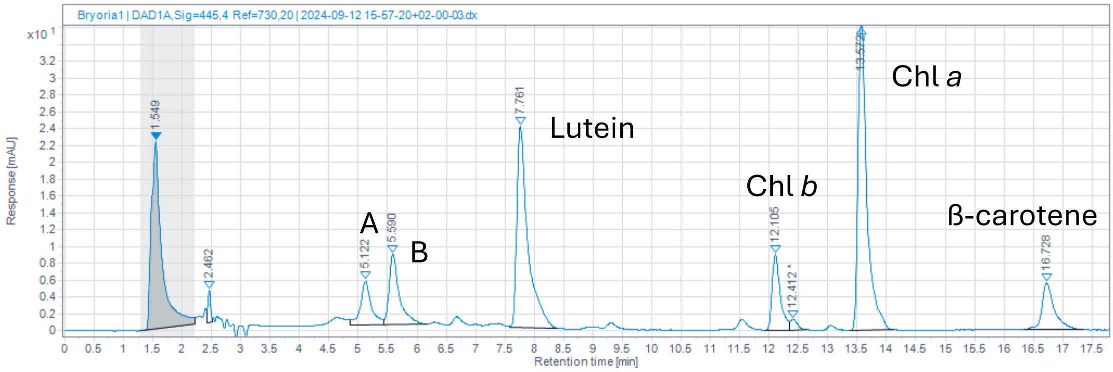

Figure S1. HPLC chromatogram of DMSO extract of dry, intact *Cetraria islandica* and *Bryoria fuscescens* thalli . In addition to the major peaks of Chl *a* and *b*, ß-carotene and lutein; smaller peaks of neoxanthin (A), violaxanthin (B) and antheraxanthin (C) are also visible.
